# Spouses’ earnings association and inequality: A non-linear perspective

**DOI:** 10.1007/s10888-022-09539-5

**Published:** 2022-04-14

**Authors:** Shoshana Grossbard, Lucia Mangiavacchi, William Nilsson, Luca Piccoli

**Affiliations:** 1 San Diego State University, HCEO and IZA, 5500 Campanile Drive, San Diego, CA 92182-4485 USA; 2 University of Perugia and IZA, Via Pascoli 20, 06123 Perugia, Italy; 3 University of the Balearic Islands, Cra. de Valldemossa km 7.5, 07122 Palma, Spain; 4 University of Trento and IZA, Via Verdi 26, 38122 Trento, Italy

**Keywords:** Assortative mating by earnings, Female labor force participation, Fertility, Inequality, Marriage, Rank dependence, J12, J22, D31

## Abstract

We analyze the association between spouses’ earnings taking account of non-linearities along both spouses’ distribution of earnings. We also document the non-linearity of the relationships between earnings and labor force participation, earnings and couple formation, and earnings and number of children. Using simulations, we then analyze how changes in spouses’ rank-dependence structure, labor force participation and couple formation contribute to the upsurge in inequality in the U.S between 1967 and 2018. We find that an increased tendency towards positive sorting contributed substantially to the rise in inequality only among dual-earner couples, while it contributed little to overall inequality across households. Temporal and distributional heterogeneity are important, as earnings association had a more substantial role in the bottom of the earnings distribution and in recent years. The decline in couple formation contributed substantially to the rise in inequality, while the increase in female labor force participation and the fertility decline had equalizing effects.

**Supplementary Information:**

The online version contains supplementary material available at 10.1007/s10888-022-09539-5.

✉ Luca Piccoli
luca.piccoli@unitn.it

Shoshana Grossbard
sgrossba@sdsu.edu

Lucia Mangiavacchi
lucia.mangiavacchi@unipg.it

William Nilsson
william.nilsson@uib.es

**JEL codes** J12 · J22 · D31

### 1 Introduction

Household income inequality has increased sharply in the United States since the 1970s (Piketty and Saez 2003; Saez and Zucman 2016). In terms of the most commonly used measure of inequality, the Gini coefficient, inequality increased by over 22% over the period 1967–2018, climbing from 0.397 to about 0.486 (Semega et al. 2020, see Table A-4). One of the commonly proposed explanations for this dramatic increase in inequality was inspired by Gary Becker's (1973, 1974) theory of marriage and attributes the increase to a rise in positive sorting by spouses' earnings. A rise in positive assortative mating has been documented in the U.S. since the 1970s both in terms of spouses' earnings (Burtless 1999; Schwartz 2010; Gonalons-Pons and Schwartz 2017) and education (Schwartz and Mare 2005; Hou and Myles 2008; Greenwood et al. 2014; Siow 2015; Eika et al. 2019; Ciscato and Weber 2020).[1] Does such increased assortative mating account for increased inequality? According to recent articles increased assortative mating has had a relatively small impact on growth in inequality: just about 1% according to Greenwood et al. (2014), 3% according to Hryshko et al. (2017) and about 5% according to both Dupuy and Weber (2022) and Eika et al. (2019).[2] Earlier studies had attributed more of the change in inequality to increased assortative mating: 10% according to Larrimore (2014), 13% according to Burtless (1999) and 25% according to Hyslop (2001).

Alternatively, a secular positive trend in female labor force participation could have been important for the evolution of inequality. Indeed, most previous studies found that the increase in female labor force participation had an equalizing effect (Cancian and Reed 1998, 1999; Cancian and Schoeni 1998; Chen et al. 2014; Grotti and Scherer 2016; Nieuwenhuis et al. 2017; Boertien and Bouchet-Valat 2020). A third explanation attributes the rise in income inequality in the U.S. to the rise in single-parent households, especially among women with low education (Iceland 2003; McLanahan 2004; Western et al. 2008). Burtless (1999) estimates that one-fifth to one-quarter of the jump in inequality in the period 1979 to 1996 was due to the sharp increase in the proportion of single-headed families. Changes in marriage rates over the period 1960 to 2005 accounted for about 18% of the rise in income inequality according to Greenwood et al. (2016) and to about 22.5% of the increase according to Larrimore (2014).

In this paper we estimate the relative contribution of four factors related to family structure—spouses' earnings association, women's labor force participation, couple formation and fertility[3]—to changes in inequality. In contrast to most previous literature on the topic, we do so

[1] According to Rosenfeld (2008) and Ghileb and Lang (2020), however, educational assortative mating would not have increased over the last decades.

[2] Greenwood et al. (2014) consider marriage sorting by education for years 1960 to 2005; Hryshko et al. (2017) consider wage-based assortative mating for years 1980 to 2009; Dupuy and Weber (2022) and Eika et al. (2019) consider educational assortative mating for the period 1962 to 2013.

[3] Recent studies estimating the cost of children and equivalence scales based on family behavioral models (Browning et al. 2013; Dunbar et al. 2013; Baudin et al. 2015; Mangiavacchi et al. 2018; Betti et al. 2020) confirm the importance of accounting for changes in family structure when analyzing inequality trends.

while taking account of temporal changes in the non-linear relationships between these factors and earnings of family members. This can be important because over the period under study a large part of the inequality action has occurred at the tails of the earnings distribution. According to CPS data (Semega et al. 2020), the income ratio for the top/bottom decile (P90/P10) increased from 9.23 to 12.60 –an increase of more than 36%–, while the P80/P20 increased only from 3.95 to 5.08, a 28.6% increase. The widening of the gap between rich and poor is confirmed by looking at income shares: the proportion of incomes held by the top 5% grew from 17.2% to 23.1% (+34%), while the income share of the lowest quintile decreased from 4.0 to 3.1% (−22.5%).

We first document that assortative mating by earnings, the relationship between earnings and labor force participation, the association between earnings and couple formation, and the relationship between earnings and fertility choices are of a non-linear nature in both 1967, our starting point, and 2018, the last year we examine.[4] In this respect, we propose a methodological innovation that consists of assessing spouses' earnings association using an extension of the rank dependence method that Dahl and DeLeire (2008) and Chetty et al. (2014) implemented in their studies of intergenerational mobility. While the rank dependence method focuses on a local average association at different income ranks, we propose to apply a similar concept to different quantiles of the earnings distribution. The resulting graph is an improvement that allows us to simultaneously highlight the non-linear rank dependence structure along both spouses' earnings distributions.

We then investigate how changes in these non-linear associations help account for increased inequality over the period 1967 to 2018. We propose a new method for evaluating the non-linear impact of changes in each factor (sorting by earnings, labor force participation, couple formation and number of children) on changes in earnings inequality over time: Monte-Carlo-like simulations of the 2018 income distribution using one-to-one matching coupled with regression predictions that capture non-linearities found in 1967. This method differs substantially from the widely used semiparametric decomposition method introduced by DiNardo et al. (1996), which is based on reweighting specific sample groups to obtain counterfactual distributions. It has more in common with Burtless' (1999) methodology to study changes in assortative mating, in which he matches spouses by rank.

To analyze the impact of each factor in its relevant population group, we analyze three increasingly general samples of working-age individuals extracted from the March Current Population Survey (CPS) data: dual-earner couples, all couples, and couples and singles. We find that in the case of dual-earner couples the increase in spouses' earnings association accounts for about 18% of the increase in inequality measured in terms of Gini coefficient. In contrast, when non-working spouses and singles are also included in the analysis, replicating the 1967 spouses' income association patterns for 2018 accounts for only about 5% of the increase in inequality over the period. When inequality is measured in terms of shares of total earnings obtained by the top 10% and the bottom 50% of the distribution, our results suggest that increased spouses' earnings association has been more relevant in the bottom half of the earning distribution, contributing almost 22% of the decrease in the bottom 50% share for dual-earner couples. In contrast, increased spouses' earnings association contributed only

[4] Even though other factors are also related to inequality, we don't include them in our models because most of the effects of these factors are captured by earnings (as is the case of assortative mating by education, given that higher education is associated with higher earnings).

about 11% of the increase in the top 10% income share. Temporal heterogeneity also proved important: it became much more relevant in recent years than it was before the nineties.

When assuming that the probability of couple formation (including marriage) in 2018 stayed the same as it was in 1967, we explain about 13% of the increase in inequality. Again, a larger portion of the change can be attributed to changes in the lower part of the income distribution and a notable temporal heterogeneity is observed. In line with the previous literature, we find that the increase in female labor force participation had an equalizing impact on inequality. Finally, the evolution in the number of children in the household, an often-overlooked factor in explaining inequality changes, played a relevant equalizing role which has been more homogeneous with respect to time and income distribution. We also show that the choice of equivalence scales is not neutral when evaluating the contribution of changes in these factors to the rise in inequality.

In interpreting these results, the reader should be aware that the simulations performed here are of a descriptive nature and are not meant to indicate causal relationships. The rest of the paper is organized as follows. Section 2 describes the empirical strategy, including data details and methods. Section 3 presents the results and Section 4 concludes.

### 2 Data and methods

#### 2.1 Data and sample selection

We use data from the 1968 and 2019 March Annual Social and Economic Supplement (ASEC) regularly added to the Current Population Survey (CPS), a monthly survey of labor force participation carried out by the Minnesota Population Center (Flood et al. 2017). Although the survey's main purpose is to collect information on employment, it also collects information on demographic status, including age, sex, race, marital status, educational attainment, and family structure. The March income supplement to the CPS contains information about income earned in the previous calendar year. The starting and ending years for our analysis are 1967 and 2018, with a 51 years gap.[5] An additional analysis splits the time span in 1992/1993.[6]

For each person who is 15 or older, the data include information on the amount of monetary income that individual earned in the preceding calendar year, including wage and salary income, business and farm income. Observations with negative earnings –less than 1% in each sample– are excluded. To compute single household equivalent earnings, we use number of members belonging to the main family as a group of two persons or more (one of whom is the householder) residing together and related by birth, marriage, cohabitation or adoption. We

[5] This is the widest viable time span before the COVID-19 pandemic outbreak, which appears to have affected the collection of 2019 data, as the number of observations is substantially smaller than 2018. Although CPS is available since 1962, most studies consider 1967 as the earliest reliable survey as the sample size increased substantially and a new processing system was introduced. In addition, prior to 1967 spouses' earning were not recorded separately.

[6] Between 1992 and 1993 a methodological change in the collection method of the ASEC-CPS sample produced a sudden jump in inequality, from 0.433 to 0.454. This is about 24% of the overall increase in the Gini from 1967 to 2018. Being artificially induced it would most certainly not be explained by any other factor considered. Coincidentally, this break is also interesting because it corresponds to when the increase in female labor force participation started to lose momentum, and eventually declined after the Great Recession. As shown in Figure A1, in 2018 that participation rate (57.4%) was about the same as in 1990 (57.5%).

select individuals between ages 30 and 55 living in couples or in single households, with or without children.[7] In 1967 couples are all married couples. In 2018 couples also include non-marital cohabitants, for by then the CPS records non-marital cohabitation.[8]

For 1967 the final sample of couples and singles consists of 21,429 households (45.8% of the original sample), the sample of all couples of 17,144 observations, and the sample of dual-earner couples of 8226 observations. For 2018 we have 30,071 households (44% of the original sample) when including both couples and singles, 19,864 couples, and 14,239 dual-earner couples.

Table 1 reports some descriptive statistics for the three samples in 1967 and 2018. For the sample of dual-earner couples the mean equivalent earnings increased by almost 87% over this period, while the Gini coefficient increased by 31%, ramping up from 0.282 to 0.370. The top 10% income share increased by 33%, from 0.218 to 0.290, while the bottom 50% income share decreased by more than 16%, from 0.306 to 0.256. Considering all couples, earnings increased by slightly less than 80%; inequality as measured by the Gini coefficient, started from a value of 0.314 and increased by more than 36%, to reach 0.428. For this sample the top 10% income share rose from 0.236 to 0.315, about 33%, while the bottom 50% declined from 0.287 to 0.216, a reduction of 25%. In the most comprehensive sample, which also includes singles, average earnings increased by just less than 65%, but the Gini coefficient, which starts at 0.360 still had a large increase of 33%, reaching 0.479 at the end of the period under study. The top 10% income share increased by almost 35% and the bottom 50% share shrank by almost 30%, down to 0.180 from 0.255. Although our samples of couples and singles have less than half the observations of the original CPS samples, the Gini coefficients are similar: slightly smaller in 1967, at 0.360 vs 0.397, and almost the same in 2018, at 0.479 vs 0.486.[9]

These statistics have been computed for equivalent earnings, using the square root equivalence scale. Compared to other popular equivalence scales (e.g. the OECD or the OECD-modified scales), which give different weights to adults and children, the square root scale allows us to simplify calculations of inequality measures in our simulations, where counterfactual scenarios imply variations in household composition, both in terms of presence of a spouse and by number of children. The choice of the square root scale is common for U.S. studies (Burkhauser et al. 2012; Larrimore 2014), but such a choice could potentially drive some of the results, as the average household size has dropped substantially over the period. Consequently, we also provide results obtained using the two extreme scales, i.e. per-capita earnings (each family member is given weight one) and household earnings (no equivalence

[7] Selection based on individual characteristics means that complex households are not dropped from the data, but only the main family within the household –the one that includes the household head as one of the spouses– is used. This has implications for the computation of household income as contributions provided by other members of the household are not included in the analysis.

[8] Unmarried couples were not identified in 1967, as such status was not recorded in the CPS, but estimates suggest that the prevalence of unmarried couples was limited to about only 1% of all couples (Fitch and Ruggles 2005). According to the 2010 census 5.3% of all couples were unmarried. Counting them as singles would have likely biased our simulation results. Our decision not to distinguish between married and unmarried cohabiting couples in 2018 is also rooted in the fact that some U.S. States recognized unmarried couples as common-law marriages (see Grossbard and Vernon 2015).

[9] For a reference, Figure A 2 plots the evolution of the Gini coefficient and average equivalized household income in 1999 dollars for the entire CPS samples of each year from 1967 to 2018. The differences can be explained because we only consider earnings by the main family, while the CPS measure considers all sources of income for all household members. The larger difference observed in 1967 is probably due to the larger proportions of complex households. This also implies that the increase in the Gini observed in our sample is larger than the one observed in CPS statistics, about 33.1% vs 23.2%.

**Table 1** Descriptive statistics

| | 1<br>Number of observations | 2<br>Mean earnings | 3<br>Gini coefficient | 4<br>Top 10% share of income | 5<br>Bottom 50% share of income |
|---|---|---|---|---|---|
| Panel A: Dual-earner couples | | | | | |
| 1967 | 8226 | 41,906 | 0.282 | 0.218 | 0.306 |
| 2018 | 14,239 | 78,332 | 0.370 | 0.290 | 0.256 |
| Percentage variation | | 86.9% | 31.1% | 33.0% | −16.5% |
| Panel B: All couples | | | | | |
| 1967 | 17,144 | 47,557 | 0.314 | 0.236 | 0.287 |
| 2018 | 19,864 | 67,403 | 0.428 | 0.315 | 0.216 |
| Percentage variation | | 79.5% | 36.5% | 33.4% | −24.9% |
| Panel C: Couples and singles | | | | | |
| 1967 | 21,429 | 35,197 | 0.360 | 0.251 | 0.255 |
| 2018 | 30,071 | 57,968 | 0.479 | 0.338 | 0.180 |
| Percentage variation | | 64.7% | 33.1% | 34.7% | −29.3% |

The mean equivalent income computed applying the square root equivalence scale to family income as the sum of individual incomes from wage and salary, business, and farm income scale is applied). We interpret the corresponding results as upper and lower bounds of the change in earning distribution attributable to changes in family structure.

### 2.2 Rank dependence analysis

Most studies of spouses' earnings association have focused on correlation measures over the entire earnings distribution (Hyslop 2001; Schwartz 2010; Hryshko et al. 2017; Gonalons-Pons and Schwartz 2017; Pestel 2017). This may have led them to overlook possible non-linearities along individual earnings distributions. Such non-linearities have been shown to be important, e.g. by Bredemeier and Juessen (2013) who found that from the 1970s to the 2000s the most pronounced increase in US wives' hours spent in the market occurred for wives of high-wage men.

To study how earnings association in couples varies along men's and women's distribution of earnings we adopt and extend the binning technique, an approach developed in the literature on intergenerational income mobility (Dahl and DeLeire 2008; Chetty et al. 2014; Bratberg et al. 2017). These articles use bins based on parents' income ranks and for each of the bins calculate the average income rank of their children. There are two main advantages of using bin means instead of correlation coefficients. First, a correlation coefficient can be zero, not capturing a non-linear dependence structure such as positive sorting in the lower part of the distribution and negative sorting in the higher part. Second, binning by rank can easily incorporate individuals with zero earnings. At the same time the binning technique also has some disadvantages: it produces discontinuous results that are similar to a scatter plot and, more importantly, it allows non-linear patterns to emerge only along the dimension of one variable. This is because the average rank of children varies non-linearly with parent's rank, but within each bin all children are averaged out. One possible solution would be to compute several quantiles of the child distribution within each bin of parents' rank, but this would reduce graphical clarity: dots' overcrowding could impede the detection of relevant patterns.

To overcome such complication, we propose a visual improvement that consists of plotting nonlinear functions that smoothen the information embedded in binned means and quantiles.

First, we analyze the rank dependence structure of spouses' earnings using a continuous mean function of one spouse's earning rank with a polynomial of degree $k$ of the partner's rank on the right-hand side, i.e.

$$r_{w,i} = \alpha + \sum_{k=1}^{K} \beta_k \, r_{h,i}^{k} + e_i, \tag{1}$$

where $r_{w,\,i}$ is wife's earning rank for family $i$, and $r_{h,\,i}$ is her husband's rank. Husband's and wife's earning ranks can be swapped. Estimating Eq. (1) by Ordinary Least Square produces a *continuous mean function*, as parameters are estimated in order to minimize the (squared) deviation from the conditional mean. The mean function is a plot of $\widehat{r}_{w,i} = \widehat{\alpha} + \sum_{k=1}^{K} \widehat{\beta}_k \, r_{h,i}^{k}$. The flexibility introduced with a polynomial allows the mean function to display a nonlinear dependence pattern along the husband's earnings rank distribution.

Second, we estimate Eq. (1) with quantile regressions (Cameron and Trivedi 2005). In contrast to OLS, estimators of quantile regressions minimize the (absolute) deviation from a given quantile of the dependent variable distribution. Such estimators allow us to produce a series of non-linear functions that are centered on different quantiles of earning rank on the left-hand side of Eq. (1). These functions show how a wife (husband) positioned at a given quantile of her (his) earnings rank distribution depends non-linearly on their husband's (wife's) rank. These *rank dependence curves* allow us to detect different rank dependence structures at different positions of each spouse's earnings rank distribution. This is visually apparent, as curves with a locally positive slope indicate a local positive earnings association; locally flat curves present no correlation; while a negative slope indicates a local negative earnings association. It is then possible that at a given spouse rank, differently ranked partners may have different earnings association, even of opposite sign. Rank dependence curves thus present a richer picture of non-linearities in spouses' earnings association.

#### 2.3 Labor force participation, marriage and number of children

Previous studies found that changes in women's labor force participation varied across husbands' income distribution (see, for instance, Cancian and Reed 1998; Bredemeier and Juessen 2013; Chen et al. 2014; Grotti and Scherer 2016). Similar patterns may be at work for marriage and number of children. As such, to establish whether there are non-linearities in the relationships between spouses' earnings, labor force participation, marriage and number of children in the household at the time of the survey, we apply standard statistical techniques. It is relevant to note that our goal here is not to identify causalities, but rather to produce reliable simulations. Thus, to let earnings capture as much variation as possible, no covariates are included in the following regressions.

We estimate the probability of not having a spouse and spouse's labor force participation (defined as probability of having a spouse with zero-income from work, business or farm) as functions of the logarithm of own income using local linear nonparametric regressions (Fan and Gijbels 1996),[10] i.e.

$$y_i = g(\mathbf{x}_i) + e_i. \tag{2}$$

Local linear regression estimates a regression similar to OLS, but a kernel function and a bandwidth assures that observations closer to the evaluation point in the distribution of the explanatory variable receive more weight. In our application, all of the values of $\mathbf{x}$ are used as

[10] More specifically, we use the *npregress* command in Stata 16.

evaluation points. This allows us to have an estimate of the relationship for each rank, and thus the resulting estimates capture any sort of non-linearity in the relationship between the outcome and the explanatory variable.

Modeling the number of children is slightly more complex because couples, single mothers and single fathers may vary in their fertility patterns. To simplify the modeling and the predictions we estimate three separate ordered probit models (Cameron and Trivedi 2005) for each household type. For single women and single men, the number of children is regressed on a cubic polynomial of earnings rank and a dummy for zero earnings. For couples, we include a cubic polynomial of both spouses' ranks and dummies for zero earnings, plus the interaction of husband's and wife's ranks.

### 2.4 Simulations

Existing research about the factors possibly contributing to the increased inequality in the U.S. has used two main methodological approaches: variance decomposition and simulations of counterfactual scenarios that are then compared to actual inequality measurements. In order to account for changes in the non-linear patterns of association between spouses' earnings, labor force participation, marriage and number of children, we follow a counterfactual approach simulating the 1967 non-linear patterns for 2018 data. The advantage of such a simulation procedure is that it makes it possible to incorporate variation along earnings distributions in the counterfactual situations being examined. This technique allows us to avoid imposing randomization (as commonly done in the existing literature) or a particular degree of association (such as a specified correlation coefficient) as the hypothetical reference case.

Inequality is often measured by Gini coefficients, and we also do so, but since changes over time may possibly be concentrated at the top or bottom of the distribution, we also use two other measures of inequality: the top 10% share of earnings, that is the share of population earnings obtained by families at the top 10% of the earnings distribution, and the bottom 50% share of earnings (Silber 1999). The details of each step of the simulation are presented in Appendix B. Here we just present the intuition behind the simulation technique.

The counterfactual simulations of the changes in the non-linear patterns of spouses' earnings association are implemented using a one-to-one matching technique, which requires equal sample sizes in both years. Since the number of observations changes substantially from 1967 to 2018 we randomly extract 200 samples from 2018, each composed of the number of observations available for 1967.[11] For example, to find a counterfactual spouse's earnings for a male in a particular rank in 2018 we search for a man at exactly the same rank in 1967 and extract his wife's rank. We then assign as the counterfactual spouse to the husband a woman who has the same rank in 2018 as the actual 1967 wife.[12] The earnings of the counterfactual spouse are then used to recalculate household earnings and it may include imputed values.

[11] This ensures that the results are not driven by a particular sample selection, as the presented results are computed taking the average of the 200 repetitions. An alternative strategy to perform a one-to-one matching without dropping observations would be to replicate all observations in each year by the number of observations of the sample of the other year. This strategy, however, would generate huge datasets for which simulations would be inconvenient. The resulting added precision of the results may not be large.

[12] For example, to assign a counterfactual spouse to a husband ranked #2654 in males' earnings distribution in 2018, we find a man ranked in exactly the same position in 1967 and find the rank of his wife, say #1896. The counterfactual wife for the 2018 husband ranked #2654 is a wife ranked #1896 in 2018. It is worth noting that this procedure fully accounts for any non-linearity that may be present in spouses' earnings association, and that rank dependence curves introduces in Section 2.2 are not used here.

This procedure is repeated for each observation of all the 200 extractions from the 2018 sample.

The procedure used to simulate the probability of having a spouse with zero earnings, of being in couple and of the number of children, is slightly different for it involves combining one-to-one matching on earning ranks with probability predictions based on the regression models proposed in Section 2.3. Even though the probability regressions are based on level of earnings, not earnings rank, the matching is still done according to rank. For instance, to simulate the counterfactual for the probability of a man having a non-working spouse in 2018 based on that probability in 1967, we substitute the predicted probability for 2018 with that of a man in the same rank in 1967.

Each of these counterfactual distributions can be included separately or jointly, given that in each simulation estimated probabilities and earnings association can be based on either 1967 or 2018. For example, if earnings association is to be evaluated, the sorting is simulated according to the 1967 patterns, but the estimations for 2018 are used to predict having a zero-earning spouse, not being in couple and the number of children. This allows us to evaluate how each single change in earnings association, labor force participation, marriage and number of children, contributes separately to inequality in 2018. When comparing the simulated earnings distribution in 2018 to that of 1967 we compare the Gini and shares of earnings of the reduced sample in 2018 to that of the actual 1967 sample and assume that the same change observed for the reduced sample (averaged across the 200 repetitions) applies to the actual Gini and shares.

## 3 Results

### 3.1 Changes between 1967 and 2018

#### 3.1.1 Association between spouses' earnings rank

Figures 1 and 2 present measures of the association between spouses' earnings distributions for samples of U.S. married couples in 1967 and 2018. Each graph presents the three different measures of such association mentioned in Section 2: (1) binned means (gray dots); (2) mean function (black line), and (3) rank dependence curves (gray lines). Even at first sight, it is apparent that spouses' earnings association patterns are highly non-linear in both 1967 and 2018. The slope of the curves, indicating local rank dependence or spousal earnings association, often changes as a function of both spouses' earnings rank.

Figure 1 (Fig. 2) plots women's (men's) earning dependence patterns as a function of men's (women's) earnings rank. In 1967, the overall correlation coefficient between spouses' earnings is close to zero (−0.04) even though the spouses' earnings ranks are locally correlated either positively or negatively at most ranks, as highlighted by the mean function (black line). The interpretation of the rank dependence curves (gray lines) is as follows. Take Fig. 1, where the y-axis represents the female rank in a global sense, i.e. it indicates the rank positions in the earnings distributions of all female spouses. The curves indicate a local ranking of spouses, each curve representing a decile. Consider for instance the curve labelled D7. The curve indicates the overall rank of women that are locally at the seventh decile of women's earning distribution for a specific men's rank. Looking at a man at men's rank 80 (on the x-axis), we know that his spouse is locally in decile 7, i.e. has a rank of 70 among the subset of spouses

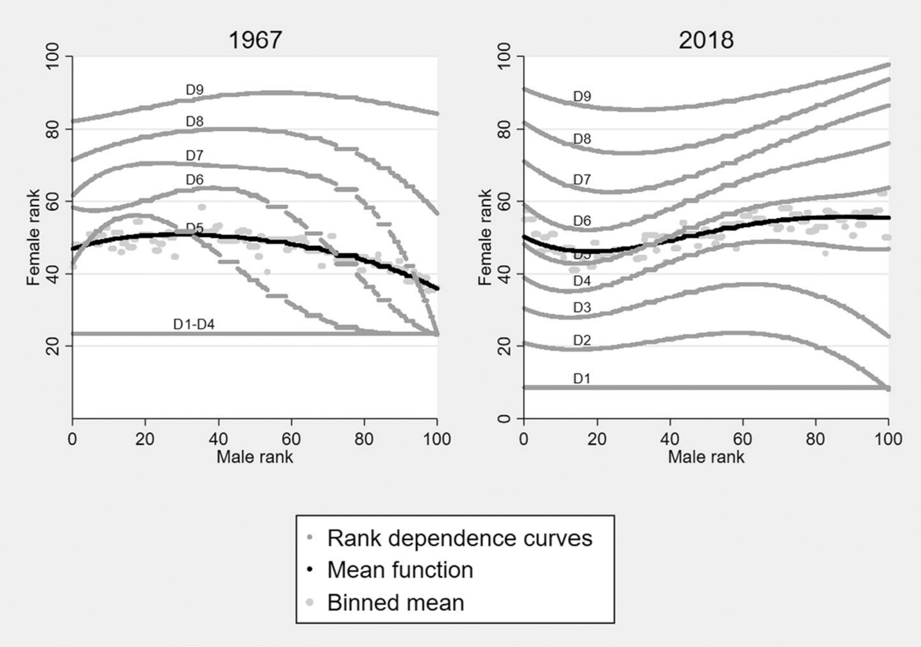

**Fig. 1** Rank dependence analysis for spouses' income association by male rank: binned means, mean function, and rank dependence curves

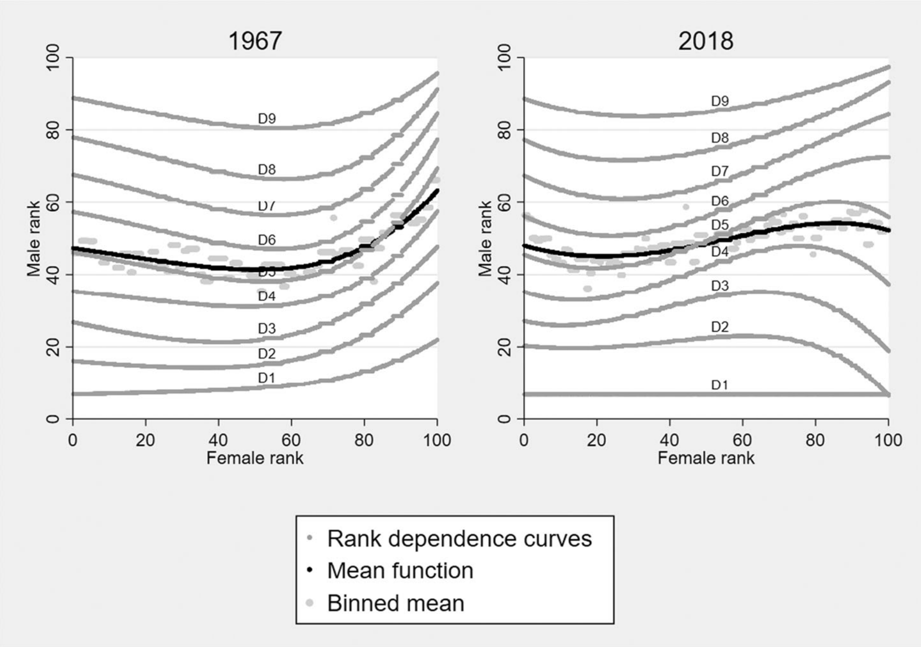

**Fig. 2** Rank dependence analysis for spouses' income association by female rank: binned means, mean function, and rank dependence curves married to men ranked around 80, and the curve gives us the corresponding global rank, i.e. about 45. The negative slope of the curve indicates that an increased male rank is associated with a reduced global spouse's rank, indicating a locally negative rank dependence structure. Now, consider women at D9 and men at rank 90. A female spouse who is locally at rank 90 is globally at rank 85. The slope of the curve is negative but rather flat, implying a locally weak negative earnings correlation

The rank dependence curves in Fig. 1 show that for rich men there is a negative association between the spouses' earnings. Negative sorting is apparent for men with an above-average earnings rank: the higher their rank the lower their wife's rank is likely to be. This negative association characterizes all couples with rich men but is least pronounced for rich men married to the richest women (e.g. women in decile D9). In contrast, for poor men, especially those in the bottom 20% of the distribution, the slopes of the rank dependence curves are positive, indicating positive sorting. As men move up in rank they are more likely to have a wife at a higher rank.[13]

In 2018 non-linearities continue to dominate the rank dependence patterns organized by male earning rank (Fig. 1, right panel). If we repeat the analysis for men in rank 80, we find that a female spouse locally at rank 90 (D9) is found globally at about rank 95, and the slope is positive. An improved male rank would increase the spouse's global ranking. For D5, we find a global rank of almost 60, but the slope is flat: an increased male rank would not improve that ranking. For D2, the spouse's global rank is about 20, but the curve has a negative slope, indicating that an increase in male rank would reduce the woman's global rank even further. These examples show that the complexity of earnings association goes far beyond what can be captured by a single average correlation coefficient.

Looking at the overall picture for 2018, rank dependence curves have positive slopes, indicating positive sorting, in the case of (a) men with earnings rank above 20 and below 60, regardless of women's rank, and (b) couples with relatively rich spouses (D6 and above). Negative sorting is found for rich men married to women with low rank (between D2 and D5). During the fifty years separating the two panels in Fig. 1, positive sorting in the lower part of men's earnings distribution has turned negative, while the negative sorting observed in 1967 for men above the median earnings translated to a more heterogeneous situation, with positive sorting among men in couple with relatively rich women and negative sorting among those married to relatively poor women.

Figure 2 is similar to Fig. 1 but takes female rank as its starting point and places it on the horizontal axis. In 1967, almost all rank dependence curves take an asymmetrical U-shape with a slightly negative association for low to medium female earnings rank (up to about rank 60). The association then turns strongly positive for higher female rank. Conditioning on female rank thus produces results that are very different from those obtained conditioning on male rank. It is apparent from Fig. 2 that in 1967 the rank dependence structure is rather homogeneous: asymmetrical U-shaped curves prevail at all ranks of the male earnings distribution except for D1, where the positive association at top female rank is somewhat weaker and no rank correlation is observed for the bottom half of women's earnings distribution.

By 2018, the right panel in Fig. 2 is remarkably similar to that of Fig. 1, suggesting a much larger degree of symmetry in the rank dependence structure between men and women. A

[13] Note that the rank of women at the bottom 40% (D1-D4) is not correlated with husband's rank as all these women are out of the labor force and have zero earnings.

negative association is observed for the first two deciles of women's earnings distribution, then the association turns positive until rank 70, where a heterogeneity similar to that observed in Fig. 1 kicks in: there is a positive association for women married to men ranked above the median, and a negative association for women married to men ranked below the median.

Overall, we observe an increase in the prevalence of positive sorting over the 50 years under investigation, a finding that has also been documented by Burtless (1999), Schwartz (2010), Gonalons-Pons and Schwartz (2017), and Eika et al. (2019). However, our results also indicate some relevant local negative sorting patterns. Pockets of negative income sorting are concentrated where (a) one partner has a very low rank; and (b) one partner is highly ranked and the other has a relatively low rank. This is observed independently of whether the spouse in the high or low earning rank is the husband or the wife. The emerging of such symmetry in the rank dependence structure in 2018 suggests that while specialization within the household (Becker 1973; Grossbard-Shechtman 1984; Grossbard 2015) is still present in some families, it has become gender neutral, almost completely overriding the traditional family model of the male breadwinner that was prevalent in 1967.

#### 3.1.2 Labor force participation

Figure 3 (Fig. 4) shows two non-linear functions for 1967 and 2018: (a) the predicted probability of a married woman (man) having zero earnings as a function of husband's (wife's) rank is indicated by a dark line; (b) the predicted probability of a man (woman) being single as a function of his (her) own rank is shown by a light line. A comparison of the darker curves on the left and right panel of Fig. 3 indicates that the relationship between wife's probability of having zero earnings and husband's rank changed drastically over these fifty years. In 1967 a married woman's probability of not having a job does not change much with husband's rank until approximately the 40th percentile of the male earnings distribution. After that point it increases rapidly with husband's rank. In contrast, in 2018 the relationship between men's rank and women's likelihood of being out of the labor force is almost flat. The drop in married women's likelihood of opting out of the labor force over the period was most striking for the wives of men in the top 4 deciles of the earnings distribution. This is consistent with recent research on opting out of the labor force being associated with women's elite education and elite universities possibly facilitating marriages involving high-income men (Hersch 2013).

Figure 4 shows the same predicted probabilities as Fig. 3, but with female rank on the horizontal axis. In 1967, husbands have a very low probability of having zero earnings and it does not vary with wife's rank. Such probability rises from close to zero to more than 10% in 2018, with an overall flat relationship with women's rank, except for the tails of the distribution, for which a positive association is observed. More specifically, the probability of having a husband with no earnings in 2018 is smaller for very low rank women (about 10%) than for very high rank women (about 20%).

The changes in rank association illustrated in Figs. 1 and 2 are likely to be related to the changes in probability that spouses earn an income documented in Figs. 3 and 4. As positive sorting has become more common at high men ranks, it also became much less common that men with high rank had wives out of the labor force. In 2018 the probability of having a non-working spouse is just slightly larger for men, and at the very top of the distribution, it is substantially equal for men and women.

The existence of pockets of negative income sorting in 2018, mostly when one spouse has a high-income rank, and the almost flat relationship between one's rank and his/her spouse

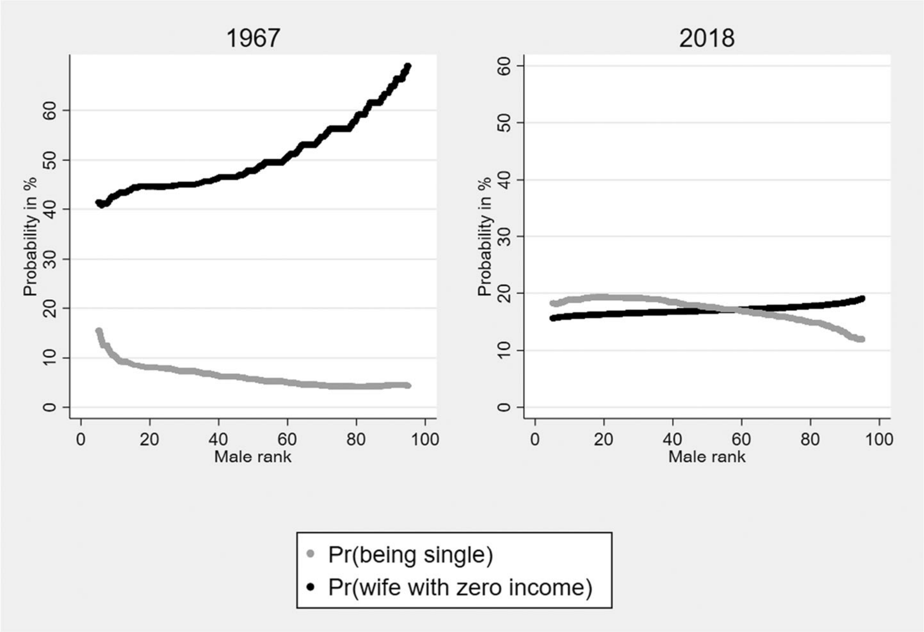

**Fig. 3** Male probability of being single and of having a wife with zero income having zero income, both suggest that specialization in intrahousehold work choices continues to be present, but that it is mostly gender neutral in 2018. This may be the result of the substantial reduction in the gender pay gap.

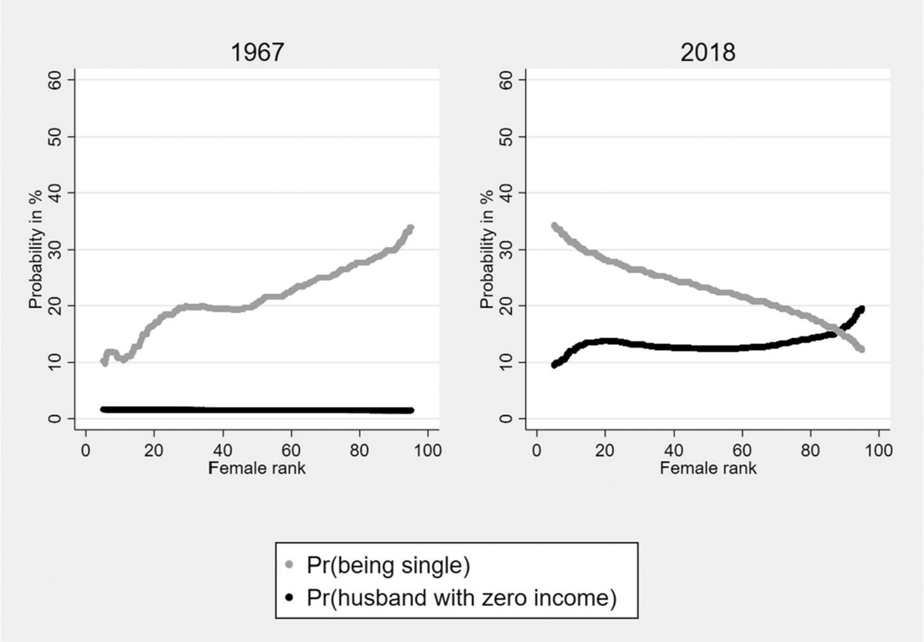

**Fig. 4** Female probability of being single and of having a husband with zero income

#### 3.1.3 Couple formation

Another variable that changed dramatically over this period is couple formation, which in both years mostly took the form of marriage. In 1967, in the age range we selected, 80% of all households were married couples.[14] By 2018 that was only the case with 63% of all households, including cohabiting couples. That is also evident from Fig. 3 and Fig. 4 in which the light color curves show how the probability of being single varied with one's own earnings rank in 1967 and 2018. Fig. 3 shows that men's predicted probability of being single was a negative function of their income rank in both 1967 and 2018, although with a slightly different shape.[15]

The change in women's probability of being single as a function of their rank is shown in Fig. 4. There was a radical switch from a strongly positive to a strongly negative relationship: in 1967 women at the highest income rank had a probability of being single that was close to 35%, versus 10% for those ranked 10 or below; in 2018 about 35% of women at the 10th percentile is single, versus only slightly more than 10% of women ranked above 90. The probability of being single thus decreases with income rank in three of the four cases we examine: men in both 1967 and 2018 and women in 2018. This contrasts with the positive slope of the likelihood of being single observed for women in 1967. This implies that as we compare 1967 and 2018, income inequality among couples may have grown because proportionally more high-income women and fewer low-income women are in couple in 2018 than in 1967.

#### 3.1.4 Number of children

Another family characteristic that changed substantially in the timespan considered here is number of children. Figure 5 plots the average number of children for all households at different ranks of the male and female earnings distribution in 1967 and 2018. The left panel highlights that there has been about a 0.8 reduction in the average number of children, with a slightly smaller reduction at lower ranks. In 1967, non-linearities play a minor role as the relationship between men's earnings and number of children follows an almost linear increasing pattern. In 2018, non-linearities are somewhat more relevant for men. We see a rather flat U-shape relationship, with the average number of children slightly larger for men at the tails of the earning distribution.

For women, the role of non-linearities in the relationship between number of children and earnings is more pronounced, both within a given year and in how it changed over time. In both years there is a similar large drop in fertility –of about 1 child– for women at the top of the earnings distribution, with the fall happening at slightly lower ranks in 2018 (at about P80) than in 1967 (P90). At lower ranks, a large drop in the number of children along these 50 years is observed, but it has not been constant along the distribution of earnings. First, the number of women with no income from work is much lower in 2018 (the relatively flat part of the line on the left part of the right panel is much shorter in 2018 than in 1967, reaching only about 20% of the sample in 2018 versus almost 50% in 1967). Second, the gap is very large, almost 1

[14] This figure does not include unmarried couples –about which no information is available–, but estimates suggest that they are about 1% of all couples.

[15] Besides the relationship between earnings and marriage, earlier literature has also focused on the changing relationship between education –a major determinant of earnings—and marriage. See, for instance, Goldstein and Kenney (2001), Fry (2010) and Torr (2011).

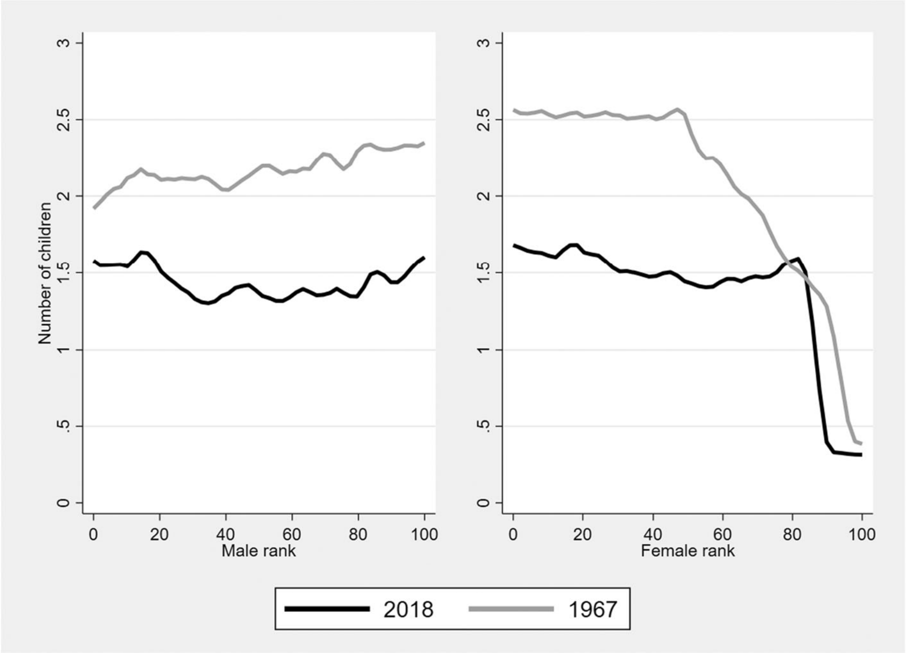

**Fig. 5** Number of children in the household across male and female income ranks child, in the middle of the income distribution. Third, in 1967 when women start to work there is a gradual reduction in fertility as the rank increases, while in 2018 a small reduction is observed when women start to work, but it remains substantially stable as earnings increase until the top of the distribution. These shifts in the relationship between fertility and women's earnings rank suggest that when using equivalized earnings as a baseline variable a direct effect of changes in the number of children on inequality could be captured in our simulation analyses.

### 3.2 What accounted for changes in inequality: a simulation analysis

#### 3.2.1 Simulation results when inequality is measured by the Gini coefficient

We have thus identified several family-related factors that could have possibly affected the evolution of inequality in the period 1967 to 2018. These factors –spouses' earnings association, spouse's likelihood of opting out of the labor force, the likelihood of being single and fertility choices– are related to spouses' earnings ranks in a non-linear way and this relationship changed notably over this timespan. The aim of the simulation analysis presented next is to analyze what inequality in 2018 would have been if the factors under analysis –and their non-linear relationship with earnings– were at their 1967 levels.

We perform simulations for three different samples of working age individuals: i) dual-earner couples, ii) all couples, including those in which one or both spouses do not work, and iii) couples and singles. We use these different samples because the simulated family factors are more relevant for specific subpopulations: i) the evolution of spouses' earnings association is more relevant for dual-earner couples; ii) to simulate the increase in female labor force participation the sample must be extended to couples in which one member does not work; and iii) to evaluate the impact of reduction in marriage (or couple formation) the sample must be further extended to include singles. In contrast, the reduction in the number of children applies to all subsamples. Thus, for dual-earner couples (Panel A of Table 2) we perform simulation models S1, which simulates earnings association between husband and wife using counterfactual earnings rank associations from 1967, and S2, which simulates 1967 number of children. In Panel B, we report simulation models for a sample that includes zero-earnings spouses. This allows us to also include counterfactuals regarding the likelihood of opting out from the labor force. Here we report results for the following simulation models: Model S3 assuming 1967 rank associations, Model S4 assuming 1967 probabilities of opting out of the labor force, and S5 assuming 1967 number of children. In Panel C we report results for a sample that includes both couples and singles. Here we report results for Models S6 to S9, adding a simulation that assumes a 1967-level probability of being single.

For each panel of Table 2 the first two rows report the Gini coefficients for the sample under analysis in 1967 and 2018 and their overall variations. The following rows report results based on simulation models: in column 1 the Gini coefficient; in column 2 the percentage change in Gini relative to the 1967 Gini; and in the last column the contribution of the simulated factor to the change in Gini coefficient between 1967 and 2018.

**Earnings association** For dual-earners couples (Panel A of Table 2) the actual Gini coefficients were 0.282 in 1967 and 0.370 in 2018. Column 2 reports the percentage change in Gini between 1967 and 2018, amounting to 31.1%. The third line reports that the Gini obtained from simulating scenario S1 "Earnings association" is 0.354. This means that if earnings association in 2018 would have followed the same non-linear patterns observed in 1967 the Gini would have increased only by 25.5%. Thus, for dual-earner couples the rise in earnings association between 1967 and 2018 contributed 17.8% of the actual Gini increase over the period.

Panel B in Table 2 reports the results for all couples, regardless of whether both spouses were in the labor force or not. Not surprisingly, the actual 1967 and 2018 Gini coefficients for this sample are substantially higher than those for the sample of dual-earner couples, amounting to 0.314 in 1967 and to 0.428 in 2018. For this sample the Gini thus increased by 36.5% over the period. When earnings association replicates the 1967 distribution (model S3) the estimated Gini in 2018 amounts to 0.418 which is 2.3% lower than the actual Gini. This implies that changes in the rank dependence of spouses' earnings contributed about 9% of the change in inequality.

Finally, Panel C reports results for the sample of couples and singles. The inclusion of singles further increases the actual Gini coefficients, to 0.360 in 1967 and 0.479 in 2018, i.e. an increase of 33.1% over the period. Simulating spouses' earnings association as in 1967 (model S6) leads to a Gini of 0.472, implying that changes in spouses' earnings association contributed 5.3% of the actual increase in Gini over the period.

Considering comparable sample selections, our results are similar to findings reported in the previous literature: Hyslop (2001) finds that in a sample of dual-earner couples assortative mating contributes about 23% of the increase in inequality; considering a sample of couples with working husbands Hrysko et al. (2017) find that changes in assortative mating contribute

**Table 2** Gini coefficients and variations for counterfactual simulation models

| | 1<br>Gin<br>Coefficient | 2<br>Change<br>ref. 1967 | 3<br>Contribution to<br>change (1967-2018) |
|---|---|---|---|
| Panel A: Dual-earner couples | | | |
| 1967 Actual Gini | 0.282 | | |
| 2018 Actual Gini | 0.370 | 31.1% | |
| *Simulated Gini in 2018* | | | |
| S1: Earnings association as in 1967 | 0.354 | 25.5% | 17.8% |
| S2: Number of children as in 1967 | 0.386 | 36.9% | −18.7% |
| Panel B: All couples | | | |
| 1967 Actual Gini | 0.314 | | |
| 2018 Actual Gini | 0.428 | 36.5% | |
| *Simulated Gini in 2018* | | | |
| S3: Earnings association as in 1967 | 0.418 | 33.3% | 8.9% |
| S4: Probability of a non-working spouse as in 1967 | 0.435 | 38.8% | −6.3% |
| S5: Number of children as in 1967 | 0.439 | 39.9% | −9.1% |
| Panel C: Couples and singles | | | |
| 1967 Actual Gini | 0.360 | | |
| 2018 Actual Gini | 0.479 | 33.1% | |
| *Simulated Gini in 2018* | | | |
| S6: Earnings association as in 1967 | 0.472 | 31.4% | 5.3% |
| S7: Probability of a non-working spouse as in 1967 | 0.479 | 33.1% | 0.2% |
| S8: Probability of being married as in 1967 | 0.464 | 28.9% | 12.7% |
| S9: Number of children as in 1967 | 0.494 | 37.5% | −13.1% |

Notes: (i) Gini coefficients are computed on the equivalized income of the reference population (ii) All Gini coefficient, except the 1967 and 2018, are simulated by Monte-Carlo techniques (see Appendix B)

about 10% of the increase in inequality; for a sample of couples and never married singles Dupuy and Weber (2022) find a contribution of about 5%. Our finding for the more comprehensive sample indicates that changes in earnings association contributed substantially less to inequality than what was reported by Burtless (1999), who also considers possible non-linearities in earning association: he found that increased earnings association contributed 13% of the increase in inequality for the whole CPS sample over the period 1979 to 1996. However, Burtless considers a time span that is substantially shorter and the corresponding increase in the Gini for that period is less than half of what we uncover (0.051 versus 0.119 points). This is also confirmed by Larrimore (2014), who also considers a non-linear spouses' earning association and finds a similar contribution (14%) to the growth of the Gini coefficient for the period 1979–89, and a very similar contribution to what we find (4%) for the 1979–2007 period. These results are also in line with our sub-period analysis reported below.

**Labor force participation** Simulation model S4 shows that if the distribution of non-working spouses had been that of 1967 (when the proportion of dual-earner couples was considerably lower) the Gini would have increased a bit more than it actually did: it would have stood at 0.435 instead of 0.428. Changes in the likelihood of opting out of the labor force thus had an equalizing effect of −6.3% to the change in Gini coefficient over the period. The figure drops to zero when couples and singles are considered (S7). This result is in line with Greenwood et al. (2016), who find no impact of female labor force participation on the inequality increase. Hyslop (2001) found a much larger and positive impact, but only for a short time span, 1979–85, a period of rapid increases in female labor force participation accompanied by relatively small increases in the Gini.[16]

**Couple formation** Simulation scenario (S8) analyzes the degree to which changes in the prevalence of couple households accounts for changes in inequality over time. The results suggest that the increase in the proportion of singles contributed substantially (almost 13%) to the increase in inequality measured by the Gini coefficient. This result is in line with findings by Burtless (1999) and Greenwood et al. (2014, 2016), although we find a smaller relative contribution to the inequality increase (they found contributions of 19.6% and 25%, respectively). Eika et al. (2019) and Dupuy and Weber (2022) found an even larger contribution of the increased proportion of singles to the increase of inequality: about 30%. One possible explanation for the discrepancy between their results and ours may be the narrower age range we consider: individuals in our sample are at least 30 years old; they are at least 25 in Burtless (1999) and Greenwood et al. (2014, 2016), and at least 26 in Eika et al. (2019) and Dupuy and Weber (2022). Given that the median age of marriage has increased substantially since the late sixties (broadly from about 23 to almost 30 years for men), it makes sense that changes in the prevalence of marriage had larger impacts on the growth in inequality reported in these other studies.

**Number of children in the household** Next, we examine how the notable reduction in number of children impacted inequality. Simulations S2, S5 and S9 in Table 2 report the results for the different samples. In the case of dual-earner couples (Panel A) the simulated 2018 Gini coefficient would be 4.3% larger than the actual 2018 Gini. This indicates that the decrease in the number of children had an equalizing effect: −19% on the increase in inequality for dual-earner couples. The equalizing effect is smaller but still relevant for the more inclusive samples: −9% for all couples and − 13% for couples and singles. The role of changes in the number of children in accounting for changes in inequality over time is comparable in magnitude to that of the increase in the prevalence of singles, but these two effects go in opposite directions.

**Equivalence scales** The use of equivalence scales is almost ubiquitous in the analysis of economic inequality, for individuals with the same income living in different-sized households are not equally well off. Thus, the use of equivalence scales helps making such individuals comparable. However, equivalence scales introduce a level of discretionality that directly affects the measurement of inequality (Cowell and Mercader-Prats 1999). The literature has proposed a number of different equivalence scales based on the demographic composition of households (Lewbel and Pendakur 2008). So far, we have used one of the simplest ones: the square root scale, which divides household earnings by the square root of household size. To analyze the sensitivity of the simulation results reported in Table 2 to the method by which equivalence scales are computed, in Table 3 we report simulations performed for the Gini coefficient with the two most extreme equivalence scales: i) no equivalence at all, i.e. simulations are conducted on total household earnings, and ii) using the household size equivalence scale, which translates household earnings to per-capita earnings.

[16] Hyslop finds that increased labor force participation of married women contributed to about 21% of the increase in inequality among couples.

Results vary substantially. Consider the Gini coefficient reported in Table 2 for dual-earner couples in 1967: the Gini is 0.282 when using the square root scale, 0.248 with no scale, and 0.346 with the per-capita scale (Table 3, panel A). This implies that this inequality measure is almost 40% larger when using per-capita versus household earnings. By 2018 the difference between the two extremes is much smaller (about 12.5%, based on a comparison of 0.358 and 0.403), due to the reduced average household size. A similar pattern is observed for the more inclusive samples, where the Gini is 22 and 27% larger with per-capita equivalence scales in 1967 and less than 10% in 2018. For the sample of couples and singles in 2018 the difference in the Gini computed with the two extreme scales is notably small, just about 3%.

Likewise, the simulation results vary substantially as a function of how equivalized earnings are computed. For example, simulation S1 leads to estimated contribution of earnings association to the Gini increase ranging from 14.8% using no scales to 24.1% using per-capita earnings. As expected, the variation drops substantially in the more comprehensive samples. The contribution to the Gini ranges from 8.2% to 10.2% in the case of all couples (Table 5, Panel B), and from 4.2% to 6.5% in the case of couples and singles (Panel C). Similar patterns are observed when analyzing labor force participation and marital prevalence. As expected, the impact that changes in fertility patterns had on inequality depends very strongly on the type of equivalence scale used. In particular, if no scale is used its impact is by definition zero, but when using per-capita earnings it has a strong equalizing effect: −58% of the increase in inequality.

**Table 3** Gini coefficients - different equivalence scales

| | 1 Gini coefficient | 2 | 3 Contribution to change (1967–2018) | 4 |
|---|---|---|---|---|
| | No scale | Per-capita | No scale | Per-capita |
| Panel A: Dual-earner couples | | | | |
| 1967 Actual Gini | 0.248 | 0.346 | | |
| 2018 Actual Gini | 0.358 | 0.403 | | |
| *Simulated Gini in 2018* | | | | |
| S1: Earnings association as in 1967 | 0.342 | 0.389 | 14.8% | 24.1% |
| S2: Number of children as in 1967 | 0.358 | 0.436 | 0.0% | −58.5% |
| Panel B: All couples | | | | |
| 1967 Actual Gini | 0.289 | 0.368 | | |
| 2018 Actual Gini | 0.418 | 0.457 | | |
| *Simulated Gini in 2018* | | | | |
| S3: Earnings association as in 1967 | 0.407 | 0.448 | 8.2% | 10.2% |
| S4: Probability of a non-working spouse as in 1967 | 0.428 | 0.461 | −8.0% | −4.6% |
| S5: Number of children as in 1967 | 0.418 | 0.479 | 0.0% | −25.7% |
| Panel C: Couples and singles | | | | |
| 1967 Actual Gini | 0.348 | 0.425 | | |
| 2018 Actual Gini | 0.490 | 0.507 | | |
| *Simulated Gini in 2018* | | | | |
| S6: Earnings association as in 1967 | 0.484 | 0.502 | 4.2% | 6.5% |
| S7: Probability of a non-working spouse as in 1967 | 0.488 | 0.511 | 1.0% | −5.0% |
| S8: Probability of being married as in 1967 | 0.466 | 0.492 | 16.8% | 18.2% |
| S9: Number of children as in 1967 | 0.490 | 0.541 | 0.0% | −40.6% |

Notes: (i) Gini coefficients are computed on the household and per-capita income of the reference population (ii) All Gini coefficient, except the 1967 and 2018, are simulated by Monte-Carlo (see Appendix B)

**Subperiod analysis** Looking at Fig. A 2, one could easily notice a large (disproportionate) jump in the Gini in the early nineties, and as it turns out, there has been a methodological change in the data collection process between 1992 and 1993 that caused an artificial jump in inequality that no factor considered in our simulations could possibly explain. Looking at CPS official Gini measures this jump is substantial. According to Semega et al. (2020), Table A-4, the Gini jumps from 0.433 to 0.454, a 5% jump that represents almost one fourth of the overall increase in the Gini between 1967 and 2018. This is also reflected in our sample of couples and singles, for which the jump in the Gini induced by the change in the data collection process is from 0.426 to 0.454, an increase of about 6% accounting for 23.5% of the overall Gini increase between 1967 and 2018. In addition, around the early nineties there has been a substantial change in some of the ongoing social trends under investigation. For instance, as shown in Fig. A 1, female labor force participation almost completely stopped growing. It even went down, possibly as a consequence of the great recession.[17] Based on the CPS, similar trends are observed for household size, which dropped at a much lower pace since the early nineties. The opposite trend, instead, is observed for never married individuals, especially women, for whom the prevalence increased much more rapidly since 1993. These considerations make the decision to split the time span around 1992–93 meaningful because some of the factors we consider may have played different roles in these subperiods.

The results presented in Table 4 refer to the sample of couples and singles and present some interesting insights. The first part of the table repeats the results obtained for the entire 1967–2018 period to ease comparisons. Interestingly, spouses' earnings association played a very minor role in the 1967–92 subperiod, just 3.2% of the Gini increase, but it gets as high as 14.5% in the 1993–2018 subperiod, indicating that in recent times earnings association is becoming increasingly important in determining inequality.

The null overall impact of the increase in female labor force participation for the whole 1967–2018 period, also hides substantial temporal heterogeneity: the strong increase in married women's employment in the first sub-period had a small equalizing effect (−5.6%), while the decrease in women's employment observed after 1993 contributed a substantial 15% to the increase in inequality.

An even stronger heterogeneity is observed for the reduction in the probability of being married. If for the whole period the increase in the number of singles has contributed 12.7% to the increase in inequality, that contribution was substantially higher in the first sub-period: adding 31.7% to the increase in inequality. In contrast, in the second sub-period the picture changes completely: the increased probability of being single provided a substantial equalizing effect of −22.2%. This may be the case because of two different trends: a boom in divorce observed in the seventies and a sharp increase in the proportion of never-married working individuals in the later period, especially among women. As suggested for instance by Ananat and Michaels (2008), the increase in the odds of divorce may lead to increased inequality and never getting married, possibly to pursue their own career, seems to have an equalizing effect.

Finally, the inequality effect of changes in number of children is more stable in the two sub-periods, although the equalizing effect has been slightly stronger in the first subperiod (−15%), when the reduction in fertility was more rapid.

[17] According to Grossbard and Amuedo-Dorantes (2007) changes in cohort sex ratios may also have contributed to drops in women's labor force participation.

**Table 4** Gini coefficients and variations for counterfactual simulation models, sub-period analysis

| | 1<br>Gini | 2<br>Change | 3<br>Contribution to |
|---|---|---|---|
| Couples and singles | Coefficient | reference | change |
| 1967–2018 | | | |
| 1967 Actual Gini | 0.360 | | |
| 2018 Actual Gini | 0.479 | 33.1% | |
| *Simulated Gini in 2018* | | | |
| S6: Earnings association as in 1967 | 0.472 | 31.4% | 5.3% |
| S7: Probability of a non-working spouse as in 1967 | 0.482 | 33.1% | 0.2% |
| S8: Probability of being married as in 1967 | 0.469 | 28.9% | 12.7% |
| S9: Number of children as in 1967 | 0.491 | 37.5% | −13.1% |
| 1967–1992 | | | |
| 1967 Actual Gini | 0.360 | | |
| 1992 Actual Gini | 0.426 | 18.4% | |
| *Simulated Gini in 1992* | | | |
| S6: Earnings association as in 1967 | 0.424 | 17.9% | 3.2% |
| S7: Probability of a non-working spouse as in 1967 | 0.430 | 19.5% | −5.6% |
| S8: Probability of being married as in 1967 | 0.405 | 12.6% | 31.7% |
| S9: Number of children as in 1967 | 0.436 | 21.3% | −15.4% |
| 1993–2018 | | | |
| 1993 Actual Gini | 0.454 | | |
| 2018 Actual Gini | 0.479 | 5.5% | |
| *Simulated Gini in 2018* | | | |
| S6: Earnings association as in 1993 | 0.475 | 4.7% | 14.6% |
| S7: Probability of a non-working spouse as in 1993 | 0.475 | 4.7% | 15.0% |
| S8: Probability of being married as in 1993 | 0.484 | 6.7% | −22.2% |
| S9: Number of children as in 1993 | 0.481 | 6.0% | −10.2% |

Notes: (i) Gini coefficients are computed on the equivalized income of the reference population (ii) All Gini coefficient, except those labelled as actual, are simulated by Monte-Carlo techniques (see Appendix B); (iii) We followed the instructions in https://cps.ipums.org/cps/income_cell_means.shtml to replace top-coded incomes with consistent cell means according to Larrimore et al. (2008) for 1992 and 1993

#### 3.2.2 Simulation results for the top 10% and the bottom 50% shares of earnings

**Sorting patterns** Table 5 reports the actual and simulated shares of earnings of the top 10% in 2018 assuming that the earnings association was that in 1967. For dual-earner couples (Panel A) the top 10% share observed in 2018 is 29% and it would have been 28.2% if earning association had been the same as in 1967. Considering that the top 10% share was 21.8% in 1967, this implies that changes in earnings association accounted for 10.6% of the increase in the top 10% share over the period. This contribution is lower than the contribution of assortative mating to the Gini, which was about 18%, indicating that the increase in spouses' earnings association may have had a larger impact on inequality for the lower part of the earnings distribution. Panel A in Table 6 confirms this intuition: earnings association contributed 22% of the reduction in the bottom 50% share. The intuition for this result can be drawn from Fig. 1. Looking at changes in earning association in the bottom 50% of men's rank, the shift toward positive association is evident for women in the bottom 50% of their distribution. In contrast, in 2018 at the top 10% of men's earnings distribution there remains a local negative association (rank dependence curves D2 to D5), while at the top 10% of women's distribution (Fig. 2) income association changed from positive at all men's ranks in 1967 to negative for men in P5 or below in 2018.

**Table 5** Top 10% income shares and variations for counterfactual simulation models

| | 1<br>Top 10% share of income | 2<br>Change ref. 1967 | 3<br>Contribution to change (1967–2018) |
|---|---|---|---|
| Panel A: Dual-earner couples | | | |
| 1967 Actual share of income | 0.218 | | |
| 2018 Actual share of income | 0.290 | 33.0% | |
| *Simulated share in 2018* | | | |
| S1: Earnings association as in 1967 | 0.282 | 29.5% | 10.6% |
| S2: Number of children as in 1967 | 0.298 | 37.0% | −12.2% |
| Panel B: All couples | | | |
| 1967 Actual share of income | 0.236 | | |
| 2018 Actual share of income | 0.315 | 33.4% | |
| *Simulated share in 2018* | | | |
| S3: Earnings association as in 1967 | 0.307 | 30.1% | 9.8% |
| S4: Probability of a non-working spouse as in 1967 | 0.320 | 35.6% | −6.6% |
| S5: Number of children as in 1967 | 0.324 | 37.2% | −11.2% |
| Panel C: Couples and singles | | | |
| 1967 Actual share of income | 0.251 | | |
| 2018 Actual share of income | 0.338 | 34.7% | |
| *Simulated share in 2018* | | | |
| S6: Earnings association as in 1967 | 0.331 | 31.7% | 8.6% |
| S7: Probability of a non-working spouse as in 1967 | 0.339 | 35.1% | −1.2% |
| S8: Probability of being married as in 1967 | 0.331 | 31.7% | 8.5% |
| S9: Number of children as in 1967 | 0.349 | 39.0% | −12.6% |

Notes: (i) Top 10% share of equivalent income is the share of equivalized income produced by the reference population owned by the richest 10% of the same population (ii) All income shares, except the 1967 and 2018, are simulated by Monte-Carlo techniques (see Appendix B)

As expected, the contribution of earnings association to growth in inequality decreases as the sample becomes more inclusive. Its role drops more for the bottom 50% income share (see Table 6, Panels B and C). For the sample of couples and singles, it accounts for only 3.5% of the reduction in the bottom 50% share, but for 8.6% of the increase in the top 10% share.

These results are comparable to those obtained by Schwartz (2010), who uses the ratio between the top 20% income share and the bottom 20% of the income share as inequality measure. Although not strictly comparable, computing a measure of inequality from our simulation defined as the ratio between the top 10% share and the bottom 50% share, we find that the increase in earnings association contributed 11.9% to the increase in this measure of inequality,[18] which is slightly larger than the finding of about 8.1% by Schwartz (2010, Table A1) for a comparable sample of couples.

**Labor force participation** The contribution of increased female labor force participation to increased inequality measured in terms of top 10% and bottom 50% shares is in line with that contribution when the Gini coefficient is used to measure inequality (Tables 5 and 6, panel B). Regardless of measure of inequality, changes in labor force participation had an equalizing effect for the sample of couples: had labor force participation remained at the 1967 levels the top 10% of the distribution would have earned an even larger share of the total (32% vs 31.5%)

[18] Although not explicitly reported in Tables 5 and 6, this measure of inequality can be computed from the ratio of the shares reported in panels B of the two tables.

**Table 6** Bottom 50% income shares and variations for counterfactual simulation models

| | 1<br>Bottom 50% share of income | 2<br>Change ref. 1967 | 3<br>Contribution to change (1967-2018) |
|---|---|---|---|
| Panel A: Dual-earner couples | | | |
| 1967 Actual share of income | 0.306 | | |
| 2018 Actual share of income | 0.256 | −16.5% | |
| *Simulated share in 2018* | | | |
| S1: Earnings association as in 1967 | 0.267 | −12.9% | 22.0% |
| S2: Number of children as in 1967 | 0.244 | −20.4% | −23.1% |
| Panel B: All couples | | | |
| 1967 Actual share of income | 0.287 | | |
| 2018 Actual share of income | 0.216 | −24.9% | |
| *Simulated share in 2018* | | | |
| S3: Earnings association as in 1967 | 0.222 | −22.7% | 8.8% |
| S4: Probability of a non-working spouse as in 1967 | 0.212 | −26.2% | −5.4% |
| S5: Number of children as in 1967 | 0.210 | −26.9% | −8.4% |
| Panel C: Couples and singles | | | |
| 1967 Actual share of income | 0.255 | | |
| 2018 Actual share of income | 0.180 | −29.3% | |
| *Simulated share in 2018* | | | |
| S6: Earnings association as in 1967 | 0.183 | −28.3% | 3.5% |
| S7: Probability of a non-working spouse as in 1967 | 0.182 | −28.9% | 1.6% |
| S8: Probability of being married as in 1967 | 0.191 | −25.4% | 13.5% |
| S9: Number of children as in 1967 | 0.171 | −32.9% | −12.2% |

Notes: (i) Bottom 50% share of income is the share of total equivalized income produced by the reference population owned by the poorest 50% of the same population (ii) All income shares, except the 1967 and 2018, are simulated by Monte-Carlo techniques (see Appendix B)

and the bottom 50% of the distribution would have a slightly lower share (21.2% vs 21.6%). When singles are also included (Panel C in Tables 5 and 6), the increase in labor force participation had a negligible impact on inequality, just around 1%.

**Couple formation** Panel C of Table 5 shows that if the probability of being married in 2018 had matched that of 1967, the share of earnings owned by the top 10% of households would be smaller (33.1% vs 33.8%), implying that the reduced probability of being in couple contributed 8.5% to the increase in the share of earnings at the highest decile. Changes in probability of being in couple contributed even more to the reduction of the bottom 50% share: about 13.5% (see Table 6, Panel C).

**Number of children in the household** Applying 1967 fertility patterns to 2018 to dual-earner couples, the equalizing effect for the top 10% share would be equal to −12.2%, and for the bottom 50% share it would be even larger: −23.1%. These effects are large magnitudes, comparable to the extent to which earnings association helps explain changes in inequality for the same sample. When looking at the other two samples–all couples and couples and singles—very similar trends are observed for top 10% share, while for the bottom 50% share the equalizing effect of reduced fertility is smaller: −8.4% for couples and −12.2% for couples and singles. This indicates that the equalizing effect of changes in number of children per household has been particularly relevant for dual-earner couples at the bottom half of the earnings distribution.

### 4 Conclusions

This paper's main goal has been to assess the degree to which changes in inequality over the period 1967 to 2018 in the United States can be attributed to changes in spouses' earnings association, labor force participation, couple formation and number of children. To measure spouses' earnings association a new graphical tool that captures non-linearity was introduced: *rank dependence curves*. This representation of earnings association is similar to what Chetty et al. (2014) used in an intergenerational mobility context, but extended to vary non-linearly along both husbands' and wives' earnings ranks. We find that spouses' earnings association patterns are highly non-linear in both 1967 and 2018, and that these patterns changed remarkably over these fifty years. Even though positive and negative sorting co-exist in both years, positive earnings associations are more common in 2018 than in 1967. It is remarkable that the rank dependence structure in 2018 is almost identical when the reference rank is that of men or that of women. This could indicate that while specialization in the intrahousehold division of work is still present in 2018, it is now mostly gender neutral.

The other family factors that could be related to the upsurge in inequality –the increase in female labor force participation, the reduction in the prevalence of couple households, and the reduction in the number of children living in the household– have a non-linear relationship with earnings ranks and changed notably over time. For instance, men's probability of having a non-working spouse in 1967 was above 40% at al ranks, with a strongly positive relationship with earnings. In 2018, the same probability is below 20% with an almost flat association. An even more striking change occurred for women's probability of being single: a strongly positive association with rank in 1967 turned into a strongly negative association in 2018.

To estimate how much each of these changes contributed to the rise in earnings inequality in the U.S. from 1967 to 2018 we compute Monte-Carlo-like simulations capturing all the non-linear relationships that emerged in the first part of the study. We find that an increased tendency towards positive sorting by earnings contributed substantially to the rise in inequality among dual-earner couples, but not when non-working spouses and singles are included. Our simulation results suggest that applying the 1967 earnings association patterns to dual-earner couples generates a Gini coefficient that is about 5% lower than the actual 2018 Gini. This implies that the increase in positive earnings association accounts for about 18% of the increase in this measure of inequality over the fifty-year period. For couples and singles the corresponding proportion of increased inequality explained by changes in earnings association amounts to only about 5%. These results are in line with the previous literature, indicating that allowing for non-linearities in earnings association has little impact on the general results. Temporal and distributional heterogeneity, however, has been substantial.

Splitting the time span around the early nineties reveals that in recent years the increasing earnings association has become a much more important factor explaining inequality in the US: it explains about 15% of the increase in the Gini for the sample of couples and singles, almost five times more than in the first sub-period 1967–1992. We also examine the contribution at the top and bottom of the earnings distribution using the top 10% and bottom 50% shares of earnings, i.e. the share of total earnings in the sample received by families placed in the top 10% or bottom 50% of the distribution. Simulations reveal that for dual-earner couples the rise in spouses' earnings association contributed twice as much to the shrinking share of the bottom 50% of the distribution than to the rising share of the top 10%: 22% versus 10.6%.

The increase in female labor force participation had an overall null effect on inequality over the whole period –just 0.2%. However, this hides substantial temporal heterogeneity: in the first subperiod, when female labor force participation rose substantially it had an equalizing effect, reducing the increase in inequality by almost 6%; in contrast, in recent years reduction in women's employment has contributed 15% to inequality.

The decreased prevalence of couple formation–including marriage and cohabitation– contributed a substantial 12.7% of the increase in the Gini coefficient between 1967 and 2018. A decreased tendency to live in couple contributed slightly more to inequality among the poorest 50%, accounting for 13.5% of the reduction in the income share of the bottom 50% but only for 8.5% of the surge in the share of the top 10%. The temporal heterogeneity in the contribution of changes in prevalence of couple formation is striking: it contributed almost 32% to the increase in inequality until the early nineties but had a substantial equalizing effect (−22%) in the following years.

Finally, the reduction in the number of children observed over these fifty years had implications for changes in inequality, namely an equalizing effect of about −13%. This effect has been slightly stronger before the nineties (−15%) than in later years (−10%) and acted in a similar way for the top 10% and bottom 50% of the distribution.

A sensitivity analysis reveals that the choice of equivalence scale has substantial effects not only on the measurement of inequality in each year, especially in 1967, but also on how each of the factors we considered helps account for changes in inequality over time. For instance, in 1967 the Gini is 0.248 using household earnings, 0.282 using the square root scale and 0.346 using per-capita earnings. Spouses' earnings association accounts for between 14.8% and 24.1% of the increase in the Gini coefficient for dual-earner couples, depending on the choice of equivalence scale. Such a choice is, of course, particularly relevant for the analysis of the number of children: its contribution varies from 0% by construction (using household earnings without equivalence scales) to an equalizing effect of almost −60% when using earnings per-capita.

Overall, we find that spouses' earnings association accounts for a limited portion of the increased inequality in the period 1967 to 2018. None of the other factors provided major explanations for increased inequality, and some had equalizing effects that compensate for the contributions of other factors to rising inequality. This implies that the main drivers of rising inequality in the US are to be found outside of factors related to the family. Increased wage inequality between workers may offer more explanatory power.

**Supplementary Information** The online version contains supplementary material available at https://doi.org/10.1007/s10888-022-09539-5.

**Acknowledgements** The authors wish to thank the extremely valuable contribution of three anonymous reviewers and the editor Philippe Van Kerm. We also acknowledge useful comments from the participants in the IZA World Labor Conference - Berlin, the Intergenerational Mobility, Gender and Family Formation in the Long Run workshop in Oslo, the annual meetings of Society of the Economics of the Household at the Paris School of Economics and in Lisbon, the Third Applied Microeconomics Workshop at the Free University of Bozen/Bolzano, the annual Meeting of Italian Society of Economic Inequality in Turin, and a seminar at University of Florence, Department of Economics and Management.

**Funding** Open access funding provided by Università degli Studi di Trento within the CRUI-CARE Agreement. William Nilsson acknowledges financial support from the Spanish Ministry of Science and Innovation grant # RTI2018-094733-B-I00.

### Declarations

**Conflict of interest** The authors have no relevant financial or non-financial interests to disclose.

**Open Access** This article is licensed under a Creative Commons Attribution 4.0 International License, which permits use, sharing, adaptation, distribution and reproduction in any medium or format, as long as you give appropriate credit to the original author(s) and the source, provide a link to the Creative Commons licence, and indicate if changes were made. The images or other third party material in this article are included in the article's Creative Commons licence, unless indicated otherwise in a credit line to the material. If material is not included in the article's Creative Commons licence and your intended use is not permitted by statutory regulation or exceeds the permitted use, you will need to obtain permission directly from the copyright holder. To view a copy of this licence, visit http://creativecommons.org/licenses/by/4.0/.

## Supplementary information

ESM 1 (DOCX 176 kb)

## Data Availability

The datasets generated and analysed during the current study are publicly available online at https://cps.ipums.org/cps/index.shtml. The guide and code to replicate the results of this study are available upon request.
