# Supplementary material for: Spouses’ earnings association and inequality: A non-linear perspective
Source: J Econ Inequal. 2022 Apr 14;20(3):611–38. doi: 10.1007/s10888-022-09539-5 (PMC9008623; doi:10.1007/s10888-022-09539-5)
Supplement: Supplementary file 1 — (DOCX 176 kb) [file 10888_2022_9539_MOESM1_ESM.docx]

## On-Line Appendices

## Appendix A: Additional Figures and Tables

Figure A 1: Female labor force participation 1967-2018 (Source: US Bureau of Labor Statistics)

Figure A 2: Evolution of the Gini coefficient and equivalized household income expressed in 1999 dollars (source CPS)

Table A 1 Evolution of household composition between 1967 and 2018

|  |  |  | 1967 |  |  |  | 2018 |  |
| --- | --- | --- | --- | --- | --- | --- | --- | --- |
| Children |  | Female singles | Male singles | Couples |  | Female singles | Male singles | Couples |
| 0 |  | 0.3915 | 0.8318 | 0.1634 |  | 0.4562 | 0.8437 | 0.2802 |
| 1 |  | 0.2037 | 0.0698 | 0.1831 |  | 0.2322 | 0.0847 | 0.2155 |
| 2 |  | 0.1681 | 0.0398 | 0.2544 |  | 0.1941 | 0.0492 | 0.3049 |
| 3 |  | 0.0981 | 0.0305 | 0.1872 |  | 0.0813 | 0.0180 | 0.1370 |
| 4 |  | 0.0653 | 0.0108 | 0.1099 |  | 0.0258 | 0.0039 | 0.0437 |
| 5 |  | 0.0313 | 0.0081 | 0.0538 |  | 0.0075 | 0.0004 | 0.0125 |
| 6 |  | 0.0212 | 0.0057 | 0.0248 |  | 0.0020 | 0.0001 | 0.0035 |
| 7 |  | 0.0108 | 0.0013 | 0.0118 |  | 0.0003 |  | 0.0014 |
| 8 |  | 0.0036 | 0.0015 | 0.0064 |  | 0.0001 |  | 0.0001 |
| 9+ |  | 0.0065 | 0.0007 | 0.0052 |  | 0.0005 |  | 0.0001 |
| Total |  | 0.1391 | 0.0616 | 0.7993 |  | 0.2141 | 0.1560 | 0.6299 |

Notes: Figures are computed using sample weights.

## Appendix B: Detailed Steps for Simulations

In the following list we detail steps of the simulations generally described in Section 2.4.

1. Choose the sample for year 2018. The sample includes males with female spouse, female with male spouse, single male and single female. The earnings variable is recoded to obtain a variable for male earnings and another for earnings of a female spouse if she is present. If she is not present earnings are zero. In the same way, a single female has the variable male spousal earnings coded to zero because no male spouse is present.

2. Separate the sample into two parts; one for male singles and male with a present spouse and another including females that are singles.

3. Use the male sample to estimate the probability to have a spouse. This is done with a local linear nonparametric regression with the command *npregress* in Stata v16. The logarithm of male earnings is used as the only explanatory variable. The model is used to obtain the predicted probability of having a spouse *at each rank* of the earnings distributions of the males. While we use a nonparametric regression technique a Probit model with a polynomial in log-earnings would produce similar results.

4. Calculate the proportion of males with zero earnings that have a spouse. This is the predicted probability for males with zero earnings (that was not included in step 3).

5. Use the male sample to estimate the probability to have a spouse with zero earnings, given that a spouse was present. Again, a nonparametric regression is specified with log-earnings as the only explanatory variable. The model is used to obtain the predicted probability of having a spouse with zero earnings *at each rank* of the earnings distribution of the males, given that a spouse was present.

6. For males with zero earnings, calculate the proportion with a spouse with zero earnings given that a spouse was present. This is the predicted probability for males with zero earnings (that was not included in step 5).

7. To take into account the number of children, we estimate three separate ordered probit models for single female, single male and couples These models are used to obtain a predicted number of children as a function of earnings.

8. Repeat steps 1-7 for year 1967.

9. Choose sample that was made in step 2 that includes single males and males with spouses and, within this group, make a random selection to obtain a sample-size equal to what is found for 1967 (for single males and males with spouses). In Table B 1 we show the one-to-one match in a table where an illustrative example is included for male rank $i=1,\ldots,n$. Note that sample weights are not used when performing the random extractions.

10. Choose a random sample from the sample of female singles. The size of the samples is selected to replicate the proportion of female singles that was found in 2018. This sample is added to the sample obtained in step 8; in Table B 1, $k$ is selected so that the proportion of single female remains the same as in 2018.

11. Mimic^[[1]](#footnote-1)^ the situation in 2018 by using the probabilities found in (3-4) and (5-6) to recode the spousal earnings to be 0. That is, for each individual ranked $i$, spousal earnings $y(Sp)_{i}$ are coded to zero if the predicted probability of not having a spouse $p(Sp)_{i}^{18}$ exceeds a random uniform draw $P_{a}\sim U\left( 0,1 \right)$, i.e. $P_{a}>p(Sp)_{i}^{18}$. The same is done for the probability to have spouse with zero earnings $p(0)_{i}^{18}$ and a random uniform draw $P_{b}\sim U\left( 0,1 \right)$. In the simulations we include the mimic spousal earnings $\hat{y}(Sp)$ according to: $\hat{y}\left( Sp \right)_{i}=y(Sp)_{i}^{18}\times1(P_{a}>p(Sp)_{i}^{18})\times1(P_{b}>p(0)_{i}^{18})$.

With this procedure it is possible that the random draws indicate positive earnings but the actual situation was to not have a present spouse, or to have a spouse with zero earnings. In these cases, actual spousal earnings must be replaced with imputed ones. $y(Sp)_{i}^{18}$ are spousal earnings that correspond to male rank $i$ in 2018, i.e. the actual spousal earnings, or the income that corresponds to the given male rank.

12. To perform the imputation of spousal earnings $y(Sp)_{i}^{18}$ when necessary, all men are sorted into 100 groups according to their position in the earning distribution. For each male a spousal earning is randomly chosen from the working spouses in his group. Notice that the imputation is only done for the cases where the probabilities indicate having a spouse with positive earnings, but the actual case was being single or having a spouse with zero earnings. The asterisk next to the rank indicator, for instance in $r_{3,*}^{18}$, indicate that those ranks of spouses are the result of imputed earnings.

13.To simulate counterfactual scenarios we substitute one of the three elements of the predicted earnings from 1967. For instance, in Table B 1 we include a counterfactual simulation where earnings association as in 1967. This amount to compute $\hat{y}\left( Sp \right)_{i}^{cf}=y(Sp)_{i}^{67}\times1(P_{a}>p(Sp)_{i}^{18})\times1(P_{b}>p(0)_{i}^{18})$, where $y(Sp)_{i}^{67}$ represents earnings of a woman chosen to have the same rank in 2018 as a woman in 1967 that is married to a man that have the same rank position in 1967 as that in 2018.

In this example we have only counterfactual assortative mating as in 1967, but we can, for example, easily change $p(Sp)_{i}^{18}$ for $p(Sp)_{i}^{67}$ to use the counterfactual situation with the probability to have a spouse as in 1967, or $p(0)_{i}^{67}$ for $p(0)_{i}^{18}$ to have the probability of a spouse with zero earnings as in 1967. Notice that if the counterfactual situation includes $p(Sp)_{1}^{67}$, the proportion of single female has to be the same as in 1967, so $k$ has to be chosen to match the 1967 proportion. $y(Si)_{l}$ with $l=1,\ldots,k$ corresponds to the earnings of female singles.

14.Assign the number of children (according to the predicted probabilities estimated in step 7). The predicted number of children depends on the male and simulated female earnings.

15. Calculate the Gini coefficient for simulated counterfactual situations using the ‘sgini’ Stata custom program^[[2]](#footnote-2)^ on the sum of male and female earnings divided by the square root of number of family members and using sample weights. Alternative equivalence scales can be applied here: dividing by 1 no equivalence scales are applied; dividing by the number of family members the per-capita scale is applied

16. Replicate steps 9-15 200 times. The average Gini from the 200 replications is finally used. To ensure that variations in the Gini coefficients are only due to the counterfactual scenario simulated and not to the resampling procedure, the counterfactual Gini is multiplied by the ratio between the Gini estimated on the original sample and that of the average mimic simulations.^[[3]](#footnote-3)^

Table B 1: Example

| 1967 | | | |  | 2018 | | | | | Mimic | Counterfactual  Assortative 1967 |
| --- | --- | --- | --- | --- | --- | --- | --- | --- | --- | --- | --- |
| Male  rank | Spouse  rank | P(Sp) | P(zero) |  | Male  earnings | Male rank | Spouse  rank | P(Sp) | P(zero) | female  earnings | female  earnings |
| 1 | $r_{1}^{67}$ | $p(Sp)_{1}^{67}$ | $p(0)_{1}^{67}$ |  | $y_{1}$ | 1 | $r_{1}^{18}$ | $p(Sp)_{1}^{18}$ | $p(0)_{1}^{18}$ | $\hat{y}(Sp)_{1}$ | $\hat{y}(Sp)_{1}^{cf}$ |
| 2 | $r_{2}^{67}$ | $p(Sp)_{2}^{67}$ | $p(0)_{2}^{67}$ |  | $y_{2}$ | 2 | $r_{2}^{18}$ | $p(Sp)_{2}^{18}$ | $p(0)_{2}^{18}$ | ${\hat{y}(Sp)}_{2}$ | ${\hat{y}(Sp)}_{2}^{cf}$ |
| 3 | $r_{3}^{67}$ | $p(Sp)_{3}^{67}$ | $p(0)_{3}^{67}$ |  | $y_{3}$ | 3 | $r_{3,*}^{18}$ | $p(Sp)_{3}^{18}$ | $p(0)_{3}^{18}$ | ${\hat{y}(Sp)}_{3}$ | ${\hat{y}(Sp)}_{3}^{cf}$ |
| 4 | $r_{4,*}^{67}$ | $p(Sp)_{4}^{67}$ | $p(0)_{4}^{67}$ |  | $y_{4}$ | 4 | $r_{4}^{18}$ | $p(Sp)_{4}^{18}$ | $p(0)_{4}^{18}$ | ${\hat{y}(Sp)}_{4}$ | ${\hat{y}(Sp)}_{4}^{cf}$ |
| … | … | … | … |  |  | … | … | … | … | … | … |
| n | $r_{n}^{67}$ | $p(Sp)_{n}^{67}$ | $p(0)_{n}^{67}$ |  | $y_{n}$ | n | $r_{n}^{18}$ | $p(Sp)_{n}^{18}$ | $p(0)_{n}^{18}$ | ${\hat{y}(Sp)}_{n}$ | ${\hat{y}(Sp)}_{n}^{cf}$ |
|  |  |  |  |  | $0$ |  |  |  |  | $y(Si)_{1}$ | $y(Si)_{1}$ |
|  |  |  |  |  | $0$ |  |  |  |  | $y(Si)_{2}$ | $y(Si)_{2}$ |
|  |  |  |  |  | … |  |  |  |  | … | … |
|  |  |  |  |  | $0$ |  |  |  |  | $y(Si)_{k}$ | $y(Si)_{k}$ |

Table B 2: Computation of the Gini coefficients for the original and simulated samples for 2018 incomes

| Sample |  | Actual^1^ | Simulation^2^ |
| --- | --- | --- | --- |
| Dual-earners | |  |  |
|  | Gini | 0.3700 | 0.3725 |
|  | S.e. | 0.0041 | 0.0038 |
| All couples | |  |  |
|  | Gini | 0.4281 | 0.4243 |
|  | S.e. | 0.0036 | 0.0021 |
| Couples and singles | |  |  |
|  | Gini | 0.4788 | 0.4720 |
|  | S.e. | 0.0033 | 0.0022 |
| 1. Computed from a 200 bootstrap repetitions on the original sample. 2. Computed from the average of the 200 sub-sample extractions from the simulations of the 2018 scenario, i.e. not imposing any factor from 1967. | | | |

1. The term `mimic’ refers to the procedure of running a simulation without applying any factor from 1967. This is done because the random extraction from the original sample coupled with the predictions from the probability estimates may generate a systematic difference in the Gini coefficient that we want to consider. [↑](#footnote-ref-1)
2. Van Kerm, P. (2009) “sgini – Generalized Gini and Concentration coefficients (with factor decomposition) in Stata,” CEPS/INSTEAD, Differdange, Luxembourg. [↑](#footnote-ref-2)
3. The procedure does not allow us to compute standard errors from the repetitions. The implementation of our simulation procedure involves passing data back and forth from Stata and GAUSS software, which is not compatible with a proper procedure to compute bootstrapped standard errors. We can still use the 200 repetitions to give a rough idea of the precision of the simulated Gini coefficients. Consider the 2018 sample of couples and singles without performing simulations. The bootstrapped standard error for the Gini coefficient is 0.0033, with a Gini of 0.4788. Now, running the simulations but without imposing any factor from 1967, the estimated average Gini coefficient is 0.4720 with a standard error of 0.0022. While we account for the difference in the Gini coefficient when presenting the simulation results, the fact the standard errors are so close suggests that the simulation procedure is unlikely to significantly compromise the precision of the estimates of the Gini coefficient. Similar results for dual-earner couples and all couples are reported in Table B 2. [↑](#footnote-ref-3)
